# Exposure to valproic acid (VPA) reproduces *hdac1* loss of function phenotypes in zebrafish

**DOI:** 10.17912/micropub.biology.000908

**Published:** 2023-09-26

**Authors:** Alec A. Jones, Terence Willoner Jr., Lacie Mishoe Hernandez, April DeLaurier

**Affiliations:** 1 Biology and Geology, University of South Carolina Aiken, Aiken, South Carolina, United States

## Abstract

Histone deacetylases are enzymes that remove acetyl groups from histone tails and are understood to act as repressors of transcriptional activity. Hdac1 has been previously shown to function in eye, pectoral fin, heart, liver, and pharyngeal skeletal development. We show that high doses of Valproic Acid (VPA) reproduce the *hdac1* phenotype. We identify *tbx5* genes as potential targets of Hdac1 in eye, pectoral fin, and heart development. Using timed exposures, we show that skeletal structures in the pharyngeal arches are impacted by VPA between 24-36 hours post-fertilization, indicating a role for Hdac1 during post-migration patterning, differentiation, or proliferation of cranial neural crest cells.

**Figure 1. Exposure to VPA produces defects in heart, eye, pectoral fin, and craniofacial development consistent with hdac1 loss of function mutants and is associated with changes to tbx5 gene expression:**
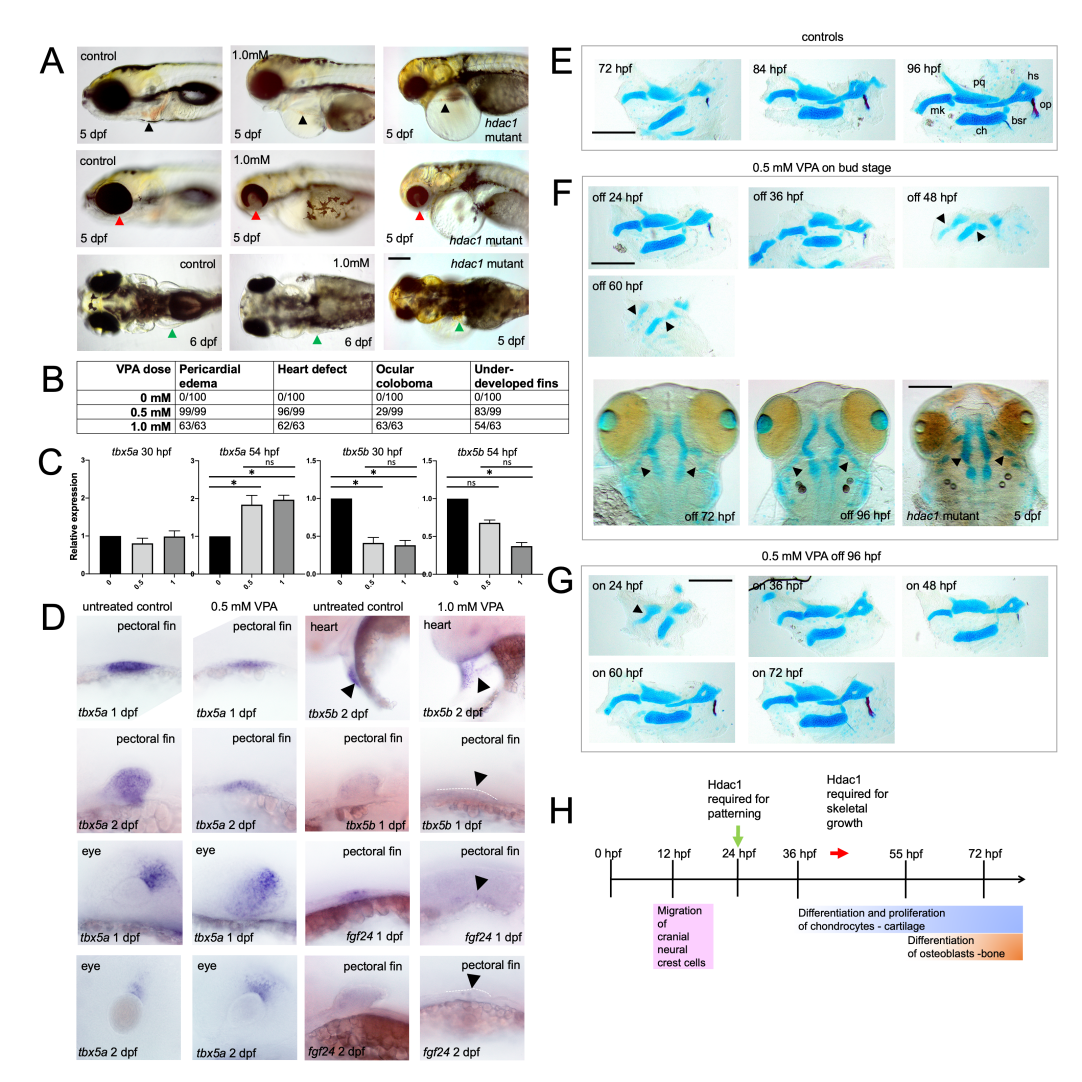
(A) Phenotypes associated with VPA treatment (0.5 and 1.0mM) compared with controls and *hdac1* mutant larvae (top row, lateral views: heart indicated by black arrow; middle row lateral views: eye indicated by red arrow; bottom row dorsal views: pectoral fin indicated by green arrow). Bar = 200 microns. (B) Frequency of phenotypes associated with specific VPA exposures (0.5 and 1.0mM) in 6 dpf larvae. (C) RT-qPCR results showing relative expression changes in *tbx5a* and *tbx5b* in VPA (0, 0.5, and 1.0mM) treated embryos compared to controls (at 30 and 54 hpf). Expression of *tbx5* genes was standardized against expression of the housekeeping gene *eif1a* and levels of expression are relative to controls. Average of 5 biological replicates and 3 technical replicates per group. Data was analyzed using a Kruskal-Wallis test to examine the effects of VPA concentration on gene expression levels. A Dunn’s multiple comparison test was used to determine statistical differences between VPA concentrations at each stage, (* p < 0.05, n.s. = not significant). Lines indicate significance between groups. (D) mRNA *in situ* hybridization showing expression of *tbx5a,* *tbx5b,* and *fgf24* in VPA treated embryos (0.5 and 1.0 mM) and controls (first and second row left columns: pectoral fin; third and fourth row left columns eyes; first row right: heart region; second through fourth row right columns: pectoral fin). Dotted lines show lateral margin of outgrowth of pectoral fin buds in VPA-treated embryos. In each experiment expression was assessed in at least 10 stained embryos and representative images are shown. (E) Skeletal preparations (lateral view) of control larvae at 72 (n = 34), 84 (n = 33), and 96 (n = 22) hpf (mk = Meckel’s; pq = palatoquadrate; hs = hyosymplectic; ch = ceratohyal; op = opercle; bsr = branchiostegal ray). Cartilage is stained blue (Alcian Blue) and bone is stained red (op and bsr, Alizarin Red). Bar = 200 microns. (F-G) Skeletal preparations of VPA-treated (0.5 mM) and *hdac1* mutant larvae where (F) VPA was added to embryos at bud stage (10 hpf) and removed at 24 (n = 49), 36 (n = 89), 48 (n = 8), and 60 (n = 59) hpf (lateral and ventral views) compared to the *hdac1* mutant; (G) VPA was added at 24 (n = 32), 36 (n = 46), 48 (n = 42), 60 (n= 46), and 72 (n = 44) hpf and removed at 96 hpf (lateral views). All VPA-treated larvae were fixed at 96 hpf, and the *hdac1* mutant larvae were fixed at 5 dpf. Cartilage is stained blue (Alcian Blue) and bone is stained red (op and bsr, Alizarin Red). Arrows are pointing to pharyngeal cartilages in ventral views. Bar = 200 microns. (H) Timeline showing events associated with pharyngeal skeleton development and hypothesized role of Hdac1. dpf = days post-fertilization hpf = hours post-fertilization

## Description

Hdac1, a class I Hdac, has previously been shown to function in regulating the cell cycle and cell proliferation, and has a critical role in development of the eye, neurons, pigment cells, craniofacial skeletal cells, and the liver in zebrafish and other organisms (Pillai et al. 2004; Stadler et al. 2005; Yamaguchi et al. 2005; Ignatius et al. 2008; Ignatius et al. 2013; Ko et al. 2019) . In craniofacial development, loss of *Hdac1* and *Hdac2 (1/2)* in mouse, and loss of *hdac1* in zebrafish is associated with severe first pharyngeal arch reduction due to loss of proliferation in cranial neural crest cells (Ignatius et al. 2008; Ignatius et al. 2013; Milstone et al. 2017) . In the case of the *hdac1 ^b382^* allele, development of the pharyngeal skeleton is described as delayed by 2-3 days, as small cartilages eventually form, whereas in the *hdac1 ^hi1618^* allele, pharyngeal cartilages are not detected even as late as 8 dpf (Pillai et al. 2004; Ignatius et al. 2008) . Skeletal reductions are reportedly not due to deficient cranial neural crest cell numbers, but rather are a defect in the behavior of post-migratory cranial neural crest cells or their derivatives once they are within the arches (Ignatius et al. 2013) . Valproic acid (VPA), a widely used anti-epileptic drug, is a known inhibitor of class I and II Hdac activity (Göttlicher et al. 2001). Previous studies show that exposure of zebrafish to VPA causes cranial neural crest cell formation defects early in development, and later differentiation defects of cells into skeletal cell types (Gebuijs et al. 2020) . In a study using timed exposures to Trichostatin A (TSA), another powerful inhibitor of class I Hdacs, Ignatius et al. (2013) concluded that the pharyngeal skeletal phenotype in zebrafish is due to a combination of an early precursor cell fate specification defect in the third to sixth posterior branchial arches but a differentiation defect in anterior first and second arches.

We observed that zebrafish embryos exposed to high doses of VPA (0.5-1.0 mM) produce a striking phenotype reproducing that observed in *hdac1* mutants, including ocular coloboma, heart chamber and looping defects, reduced pectoral fin outgrowth, and pharyngeal skeletal defects. The combination of defects in eye, heart, and pectoral fin led us to examine changes in expression of co-orthologs *tbx5a* and *tbx5b* , which are expressed in these tissues during embryonic development (Albalat et al. 2010) , and where loss of function is reported to cause defects in eye, heart, and pectoral fins (Garrity et al. 2002; Parrie et al. 2013; Pi-Roig et al. 2014; Boyle Anderson and Ho 2018) . Because the eye, heart, and pectoral fins are specifically impacted by VPA exposure and in *hdac1* mutants, we hypothesize that inhibition of Hdac1 by VPA leads to changes in expression of *tbx5* genes, which impacts development of these structures. We further examined the role of VPA exposure on craniofacial development with a goal of determining the window of sensitivity of cranial neural crest cells and their derivatives to inhibition of Hdac1 function. We hypothesize that post-migratory cranial neural crest cells are most sensitive to VPA treatment during periods when cells are populating the pharyngeal arches, proliferating, and differentiating into skeletal cell types. Overall, this study shows that higher doses of VPA than previously shown produce phenotypes most consistent with Hdac1 loss of function. Furthermore, VPA treatment leads to upregulation of *tbx5a* in retina and downregulation of *tbx5b* in pectoral fins and heart, potentially indicating a mechanism of action of Hdac1 in formation of these structures. Lastly, VPA treatment is associated with impacts to craniofacial cells, specifically during 24-36 hpf when neural crest cells are undergoing proliferation and differentiation into skeletal cells.

**Exposure to VPA produces phenotypes consistent with *hdac1* mutants:** Treatment with VPA produced defects including a “heartstrings” (Garrity et al. 2002) phenotype (A, top row indicated by black arrows; B) also seen in *hdac1* mutants, where the chambers of the heart failed to form, heart looping was absent or weak, and edema of the heart chamber was present. Ocular coloboma (A, middle row indicated by red arrows; B), caused by defective closure of the eye fissure (Lingam et al. 2021) , was also detected in VPA-treated and *hdac1* mutant larvae. Pectoral fins were reduced in VPA-treated and *hdac1* mutant larvae (A, bottom row indicated by green arrows; B), appearing as a small outgrowth on the side of the body compared to control fins at the same stage. The frequency (B) of edema and heart defects were similar at 0.5 and 1.0 mM VPA dosages (0.5 mM = 100% edema and 97% heart defect; 1.0 mM = 100% edema and 98% heart defect) whereas frequencies of eye and pectoral fin deficiencies were lower at the lower dosages (0.5 mM = 29% ocular coloboma and 84% pectoral fin; 1.0mM = 100% ocular coloboma and 86% pectoral fin). Overall, these findings show that treatment with VPA at doses at or above 0.5 mM can reproduce *hdac1* mutant phenotypes, expanding the range of tools for manipulating Hdac1 activity in zebrafish, such as in timed-exposure treatment experiments.

**Expression of *tbx5a* and *tbx5b* are changed significantly in a dose-dependent manner with VPA exposure:** Analysis of VPA treated fish with RT-qPCR to detect *tbx5a* and *tbx5b* show significant dose-dependent down regulation of *tbx5b* and upregulation of *tbx5a* (C). At 30 hpf, *tbx5a* expression is not significantly different in treated vs. control embryos. At 54 hpf, *tbx5a* expression is significantly higher in embryos treated with 0.5 and 1.0 mM VPA compared to controls (p < 0.05). At 30 hpf, *tbx5b* expression is significantly lower in embryos treated with 0.5 and 1.0 mM VPA compared to controls (p < 0.05), and at 54 hpf *tbx5b* expression is significantly lower in embryos treated with 1.0 mM VPA compared to controls (p < 0.05). To validate RT-qPCR results, mRNA *in situ* hybridization was performed on embryos at stages equivalent (1 and 2 dpf) to samples used for RT-qPCR (D). Because RT-qPCR showed significant changes in *tbx5a* with VPA used at 0.5 mM doses and *tbx5b* with VPA used at 1.0 mM doses, mRNA *in situ* hybridization was performed on embryos treated at the same doses. In general, mRNA *in situ* hybridization results support RT-qPCR findings. In the pectoral fin bud, *tbx5a* is expressed at equivalent intensity in untreated control and VPA-treated embryos at 1 and 2 dpf, although in treated embryos the fin bud is substantially reduced in size compared to controls. In the retina, *tbx5a* expression is expanded ventrally in VPA-treated embryos at 1 dpf in VPA-treated embryos compared with controls and shows a broader lateral expression pattern in treated samples at 2 dpf. Expanded retinal *tbx5a* expression in treated embryos, in the absence of increased heart or pectoral fin expression, could explain the significant increase of *tbx5a* expression detected by RT-qPCR in treated embryos. The function of *tbx5a* in zebrafish retinal patterning is not well-understood, although studies in chick show that *Tbx5* acts to maintain dorsal retinal identity (Koshiba-Takeuchi et al. 2000) . The results shown here suggest a potential role for *tbx5a,* or a pathway involving *tbx5a,* as a target of Hdac1, where inhibition of Hdac1 potentially causes upregulation of *tbx5a* , leading to abnormal retinal patterning. In contrast to *tbx5a* upregulation, *tbx5b* expression is downregulated in VPA-treated hearts at 2 dpf, and in pectoral fin at 1 and 2 dpf. Expression of *tbx5b* was not detected in the eye of either control or VPA-treated embryos in our experiments. A target of *tbx5a* and *tbx5b* in pectoral fin development is *fgf24* , which is expressed in ectoderm and underlying mesoderm of the fin bud, and functions to initiate and sustain pectoral fin outgrowth (Pi-Roig et al. 2014; Boyle-Anderson et al. 2022) . In both *tbx5a* mutants and *tbx5b* morpholino knock-downs, *fgf24* expression is absent or reduced in the pectoral fin buds (Pi-Roig et al. 2014; Boyle-Anderson et al. 2022) . In our case, the reduction of *tbx5b* expression rather than *tbx5a* , which is expressed normally in treated embryos, may explain the reduction of *fgf24* expression in fin buds. Although *tbx5* genes have not previously been identified as targets of Hdacs, our observations support a hypothesis that *tbx5b* is positively regulated by Hdac1, and controls pectoral fin and heart development. Furthermore, our observations support a hypothesis that *tbx5a* is not a target of Hdac1 in the pectoral fin and may be negatively regulated by Hdac1 in the eye, where changes to *tbx5a* expression underlie eye defects associated with loss of Hdac1 function.

**Timed exposures to VPA indicates an interval of sensitivity of pharyngeal skeletal cell types to Hdac inhibition:** To initially establish the temporal window of sensitivity of cranial neural crest cells and their derivatives to VPA, experiments were conducted exposing embryos to VPA at intervals of 10 (bud stage)-24 hpf, 10-36 hpf, 10-48 hpf, and 10-60 hpf (F). In larvae treated from 10-24 hpf (F, top left), skeletal development was delayed compared to controls (E), although individual cartilage and bone element structures appeared normal at 96 hpf. A similar pattern of delay was observed in larvae treated from 10-36 hpf (F, top middle), although individual cartilages and bone were smaller and more malformed than in 10-24 hpf exposures. In larvae treated 10 hpf up to 48 (F, right top), 60 (F, left middle), and 72 hpf (F, bottom left), cartilages were severely affected. The Meckel’s, palatoquadrate, and ventral portion of the hyosymplectic cartilage were small, malformed, and there was a lack of separation of elements between Meckel’s and palatoquadrate cartilages, and ceratohyal and hyosymplectic cartilages (indicated by arrows) in larvae treated for 10-48 and 10-60 hpf, compared to controls (E). In fish treated for 10-72 hpf (F, bottom left) and up to 10-96 hpf (F, bottom middle), skeletal elements were very rudimentary and phenocopied *hdac1* mutants at 5dpf (F, bottom right; indicated by arrows). Ventral whole mounts are shown here as lateral flat mount preparations of these specimens could not be performed. To refine the temporal sensitivity of cells to VPA, experiments were conducted exposing embryos and larvae to VPA at intervals of 24-96 hpf, 36-96hpf, 48-96 hpf, 60-96 hpf, and 72-96 hpf (G). Larvae treated from 24-96 hpf (G, top left) showed the most severe phenotypes, with small and malformed cartilages, no bone formation, and a lack of separation between the Meckel’s and palatoquadrate cartilages. Later-onset exposures did not show such severe phenotypes, and cartilages were mostly normally shaped (G). Delays in growth but not patterning were apparent in larvae treated 36-96 hpf (G, top middle) and 48-96 hpf (G, top right), although larvae treated for 60-96 hpf (G, bottom left) and 72-96 hpf (G, bottom middle) show only very slight delays (i.e. lack of bone compared to controls). In conclusion, results of these experiments point to the 24-36 hpf window as a key interval that post-migratory cranial neural crest cells are sensitive to Hdac1 reduction (H). Fish exposed to VPA prior to 24 hpf recover skeletal patterning, indicating that the phenotypic effects caused by prolonged exposure to VPA treatment or *hdac1* loss is due to events that impact neural crest-derived cells, subsequent to their migration into pharyngeal arches. Events impacted by VPA and/or *hdac1* loss could include defects to proliferation, differentiation, and patterning of cells within the pharyngeal arches, and defects in skeletal tissue formation. Given the result that larvae can somewhat recover development from VPA if removed by 24-36 hpf, and that VPA treatment from 36 hpf onward has limited effect on skeletal patterning, we hypothesize that the critical period of requirement for Hdac1 on skeletal development is after 24 hpf, and before or around 36 hpf. During this period neural crest cells undergo dorsal ventral patterning, which leads to identity of structures, suggesting that VPA disrupts normal patterning cues in neural crest or non-neural crest cell types within the pharyngeal arches. Exposure of larvae to VPA after 36 hpf does not produce patterning defects, but cartilages and bone are smaller, and this is relative to the duration of exposure (i.e. longer VPA exposure causes more reduced growth of elements). This leads us to the hypothesis that Hdac1 has a secondary, later effect on development of skeletal structures post-patterning which may be related to cell proliferation, differentiation, and/or growth of structures. Although this study did not investigate cellular processes, failure of cells to exit the cell cycle and failure to switch from proliferation to differentiation have been documented in *hdac1* mutant retina, and these processes may also be involved in pharyngeal skeletal development (Stadler et al., 2005; Yamaguchi et al., 2005) . To better understand the temporal role for Hdac1 on pharyngeal development, future studies will investigate effects of VPA and *hdac1* loss on expression of axial patterning genes (e.g. *dlx, hand2* ) (Talbot et al. 2010) , and also markers associated with skeletal cell differentiation, and skeletal element growth (e.g. *sox9* , *runx2b, col1a2, osx* ) (Flores et al. 2006; Eames et al. 2012; Kague et al. 2016) .

## Methods

*Zebrafish husbandry and generation of embryos:* Wild type AB and heterozygous *hdac1 ^b382^* zebrafish were reared and maintained at 28.5 degrees Celsius on a 14 hour on/10 hour off light cycle. *hdac1 ^b382^* zebrafish are maintained as heterozygotes as homozygotes die by 6 or 7 dpf. Fish were fed a standard diet and were maintained following guidelines from ZIRC and the Zebrafish book (Westerfield 2007) . Embryos were reared in E2 embryo media alone (Westerfield 2007) , or in E2 media containing 1-phenyl-2-thiourea (Acros Organics) to inhibit pigment formation for mRNA *in situ* hybridization (Westerfield 2007) . Embryos were anesthetized using buffered MS-222 (MP Biomedical) (Westerfield 2007) and for fixed in 2-4% PFA/1X PBS at 1-6 dpf for further analysis. The *hdac1 ^b382^* line was genotyped as described previously (Ko et al. 2019) . All procedures were approved by the Institutional Animal Care and Use Committee (IACUC) of the University of South Carolina Aiken.

*VPA treatment:* To determine the window of sensitivity of craniofacial cells to loss of Hdac1 function, we performed experiments using VPA (Tocris, cat. no. 2815, Batch no. 1A/217334) at doses 0.5 mM and 1.0 mM. VPA was diluted into E2 embryo media and was applied to zebrafish embryos at between 10 hpf -5 or 6 dpf (A and B), 10 hpf - 1-2 dpf (C and D), and at intervals between 10-96 hpf (F and G). In each experiment, at least 100 embryos were exposed to VPA in each treatment, although final numbers scored are shown in Figure 1 (E-G). After VPA exposure, larvae were washed 3X and allowed to develop in normal embryo media until fixation.

*mRNA extraction and RT-qPCR:* Total mRNA was extracted from batches of 25 control and VPA-treated embryos at 30 and 54 hpf using TRIzol reagent (Ambion), followed by isopropanol/ethanol purification and DNAse I treatment (New England Biolabs). To quantitatively assess any fold changes in *tbx5a* or *tbx5b* expression in the treated embryos, triplicate RT-qPCR reactions were performed using primers for *tbx5a* , *tbx5b* and *eif1a* (control) using 3 μl of RNA (750 ng total) from each of the samples (triplicates of treated and untreated embryos), 10 μl Luna ® Universal One-Step RT-qPCR master mix (New England Biolabs), 1 μl of Luna ® WarmStart RT enzyme mix (New England Biolabs), 0.8 μl of each forward and reverse primer (from 10 μM stock), and 4.4 μl water. Reactions were cycled under the following conditions: 55 °C for 10:00 min., 95°C for 5 mins., followed by 40 cycles of 95°C for 10 s and 60°C for 1:00 min (Biorad C1000 Touch ^TM^ Thermal Cycler, CFX96 ^TM^ Real-Time System). Primers for *tbx5a* , *tbx5b* , and *eif1a* were described previously (Parrie et al, 2013) . In total, 5 biological replicates and 3 technical replicates were performed per group. Changes in *tbx5a* and *tbx5b* expression were standardized to expression of the housekeeping gene *eif1a* . Levels of gene expression for controls were set to 1.0, and results show the relative change of gene expression in treatment groups compared to controls. Data was analyzed using a Kruskal-Wallis test to examine the effects of VPA concentration on gene expression levels. A Dunn’s multiple comparison test was used to determine statistical differences between VPA concentrations at each stage.

*mRNA in situ hybridization:* Digoxigenin (DIG)-labeled RNA probes for *tbx5a* , *tbx5b, and fgf24* expression were synthesized using templates as previously described (Garrity et al. 2002; Bhat et al. 2013; Parrie et al. 2013) . mRNA *in situ* hybridization was performed to determine tissue-specific differences in in VPA treated embyros at 1 and 2 dpf using standard *in situ* methodology (Thisse and Thisse 2008) . Briefly, treated and untreated embryos were fixed in 4% PFA/1XPBS, rinsed with MeOH/1xPBS washes and stored at -20 degrees Celsius until use. For *in situ* , embryos were rehydrated into 1XPBS, permeabilized with 10 microgram/ml Proteinase K, followed by 4% PFA fixation and 1x PBS washes. Embryos were incubated in prehybridization solution for 1-4 hours at 70 degrees Celsius and 50 to 100 ng of DIG-labeled probes were added to embryos in hybridization buffer and incubated overnight at 70 degrees Celsius. On the second day, embryos were washed in a series of 0.1-5x SSC, formamide, Tween-20 (Fisher) and 1X PBST solutions and incubated overnight with 1:1000 anti-DIG-AP antibody (Roche). On the third day, embryos were washed in NTMT buffer, followed by coloration with NBT/BCIP (Roche) in NTMT.

*Alcian Blue and Alizarin Red staining:* Alcian Blue and Alizarin Red staining to label cartilage and bone was performed as described (Walker and Kimmel 2007) .

*Imaging and Statistical analysis:* All samples were flat mount preparations and imaged using an Olympus BX41 compound microscope and cellSens software. Statistical analysis and graphs were generated using GraphPad Prism software.

## Reagents

**Table:** 

Strain	Genotype and ZFIN ID	Source
* hdac1 ^b382^*	Chr 19: 29812782 (GRCz11) T/G transversion in intron flanking exon 5, creating a new splice acceptor site ZDB-GENO-071220-28	Established mutant line previously published (Nambiar et al. 2007)
